# Detoxification of Corncob Acid Hydrolysate with SAA Pretreatment and Xylitol Production by Immobilized *Candida tropicalis*

**DOI:** 10.1155/2014/214632

**Published:** 2014-07-15

**Authors:** Li-Hong Deng, Yong Tang, Yun Liu

**Affiliations:** ^1^MOE Key Laboratory of Wooden Material Science and Application, Beijing Key Laboratory of Lignocellulosic Chemistry, Beijing Forestry University, Beijing 100083, China; ^2^Beijing Key Laboratory of Bioprocess, College of Life Science and Technology, Beijing University of Chemical Technology, Beijing 100029, China

## Abstract

Xylitol fermentation production from corncob acid hydrolysate has become an attractive and promising process. However, corncob acid hydrolysate cannot be directly used as fermentation substrate owing to various inhibitors. In this work, soaking in aqueous ammonia (SAA) pretreatment was employed to reduce the inhibitors in acid hydrolysate. After detoxification, the corncob acid hydrolysate was fermented by immobilized *Candida tropicalis* cell to produce xylitol. Results revealed that SAA pretreatment showed high delignification and efficient removal of acetyl group compounds without effect on cellulose and xylan content. Acetic acid was completely removed, and the content of phenolic compounds was reduced by 80%. Furthermore, kinetic behaviors of xylitol production by immobilized *C. tropicalis* cell were elucidated from corncob acid hydrolysate detoxified with SAA pretreatment and two-step adsorption method, respectively. The immobilized *C. tropicalis* cell showed higher productivity efficiency using the corncob acid hydrolysate as fermentation substrate after detoxification with SAA pretreatment than by two-step adsorption method in the five successive batch fermentation rounds. After the fifth round fermentation, about 60 g xylitol/L fermentation substrate was obtained for SAA pretreatment detoxification, while about 30 g xylitol/L fermentation substrate was obtained for two-step adsorption detoxification.

## 1. Introduction

Increasing attention has been paid on xylitol production from corncob hydrolysate in China because corncob is widely distributed in northern, central, and southern China with more than 40 million tons annually [1]. Compared to chemical process, fermentation production of xylitol from corncob acid hydrolysate is an attractive and promising alternative owing to environmentally friendly merit [2]. However, acid hydrolysis corncob cannot be directly used as fermentation substrate owing to various inhibitors, which would inhibit microbial growth and enzyme activity. Among these inhibitors, acetic acid and phenolic and furfural compounds are particularly predominant. It was demonstrated that acetic acid was derived from acetyl compounds during solubilization and hydrolysis of hemicellulose [3, 4]. Phenolic compounds were formed from lignin degradation during acid hydrolysis [5]. Furfural, the dehydration product of pentoses, was commonly found in hemicellulose hydrolysis [6]. Pereira et al. [7] reported that the inhibitors of acetic acid, ferulic acid, and syringaldehyde in fermentation substrate would affect microbial cell growth, xylose consumption, and xylitol yield during microbial fermentation for xylitol production. The toxicity of acetic acid was more severe under low pH conditions than under high pH conditions [8, 9]. Duarte et al. [6] reported that phenolic and furfural compounds had strongly detrimental effect on specific growth rate of microorganism and biomass productivity. Because of these negative impacts of inhibitors on microbial metabolism, it is necessary to exploit a detoxification process of acid hydrolysate prior to microbial fermentation for xylitol production [9].

The detoxification of acid hydrolysate, including biological, physical, and chemical methods and their combination processes, has been intensively available in literatures [6, 10, 11]. The processing of adsorption with activated charcoal has been commonly considered a promising technology to reduce inhibitors content [2]. In addition, monitoring the hydrolysis conditions could control the inhibitors formation to some content [12]. It is well known that mild alkaline pretreatment can remove lignin with little impact on hemicellulose content [13]. Soaking in aqueous ammonia (SAA) has been demonstrated to be a promising pretreatment method of delignification and increase the accessibility of hydrolytic enzymes to cellulose [14]. In our previous work, SAA pretreatment was employed to enhance the fermentation efficiency of rice straw acid hydrolysate for xylitol production [15]. However, it has not been reported so far on the effect of SAA pretreatment detoxification and adsorption detoxification on behavior of xylitol production by repeated-batch fermentation from corncob hydrolysate by immobilized whole cell of* C. tropicalis*.

Therefore, in this work the effects of SAA pretreatment on the content of cellulose, hemicellulose, and lignin of corncob were investigated. Then, SAA pretreatment and the traditional two-step adsorption detoxification affecting the inhibitors detoxification efficiency and sugar contents variance in the acid hydrolysate corncob were comprehensively compared. Finally, the kinetic behaviors and operational stability were evaluated for xylitol fermentation production by immobilized whole cell of* Candida tropicalis* from corncob acid hydrolysate through SAA detoxification.

## 2. Material and Methods

### 2.1. Material

The corncob particles (3~5 mm) were bought from Chifeng, Inner Mongolia, China. The corncob was air-dried and stored at room temperature in a sealed plastic bag. Strain* Candida tropicalis* As2.1776, was bought from the Institute of Microbiology, Chinese Academy of Sciences, was maintained at 4°C in agar slant medium containing glucose 1.0%, yeast extract 0.5%, peptone 1.0%, and agar 2.0%.

### 2.2. Soaking in Aqueous Ammonia (SAA) Pretreatment

30 g corncob particle was pretreated with 300 mL (w/v) aqueous ammonia in a 1-L screw-capped Pyrex laboratory bottle. The corncob was soaked for hours without agitation. Three crucial parameters affecting detoxification efficiency, such as temperature (30, 50, 70, and 90°C), ammonia concentration (2.5, 5.0, 7.5, 10, and 12.5%), and soaking time (2, 4. 6, 8, and 10 h), were comprehensively investigated in this work. After SAA pretreatment, the treated corncob particle was collected through filtration with Whatman filter paper (20–25 *μ*m pore size) using a vacuum filtration setup and rinsed with distilled water to neutral pH. Water-rinsed particle was air-dried at 105°C and stored in sealed plastic bags at 4°C until use. The corncob solid recovery was determined according to the differential weight before and after SAA pretreatment.

### 2.3. Comparison of Hydrolysis and Detoxification Efficiency of Corncob Treated by SAA Pretreatment and Traditional Two-Step Adsorption Method

3 g of corncob particle was pretreated by SAA at 70°C for 10 h using 10% ammonia aqueous solution. Then the pretreated corncob was hydrolyzed with 1% H_2_SO_4_. The hydrolysate was adjusted pH to 7.0 and concentrated with vacuum at 80°C. Finally, the concentrated hydrolysate of corncob with SAA pretreatment was employed to determine the sugars and inhibitors content with HPLC and UV-vis spectrophotometer analysis. Acid hydrolysis rate of xylan was calculated according to (1); the acid hydrolysis of xylan in corncob without SAA pretreatment was carried out as blank control. Consider

(1) Hydrolysis  rate  of  xylan(%) =(Total  xylose  and  arabinose(g)in  hydrolysate×0.9)Xylan(g)in  corn  cob  material.

For traditional two-step absorption detoxification, 3 g of corncob was directly hydrolyzed with 1% H_2_SO_4_ without SAA pretreatment. After acid hydrolysis, the hydrolysate was adjusted to pH 7.0 with Ca(OH)_2_ and then filtered and concentrated with vacuum at 80°C. In the first step detoxification, the concentrated acid hydrolysate was adjusted to pH 3.0 with absolute H_2_SO_4_ and adsorbed with 4% (w/v) activated charcoal. Following the first step detoxification, the acid hydrolysate was successively adjusted to pH 9.0 with Ca(OH)_2_ and adsorbed with anionic exchange resin 201 × 4 (2 g resins 10 mL^−1^ hydrolysate) in the second step detoxification. After two-step adsorption detoxification, liquid aliquots were withdrawn to determine the sugars and inhibitors content with HPLC and UV-vis spectrophotometer analysis. Acid hydrolysis rate of xylan was estimated according to (1).

### 2.4. Microbial Inoculum Cultivation and Whole Cell Immobilization

The inoculum culture media of strain* C. tropicalis* As2.1776 were composed of D-xylose 10 g/L, glucose 10 g/L, yeast extract 10 g/L, KH_2_PO_4_ 5 g/L, and MgSO_4_ *·*7H_2_O 0.4 g/L. After inoculum, the strain was cultivated in 250 mL Erlenmeyer flasks containing 50 mL of culture media on a rotatory shaker at 30°C and 200 rpm for 22 h. The broth was centrifuged to collect microbial cell for immobilization. The ratio of dry cell to immobilization carrier was 5.86 : 1000 (w/w), and the carrier was composed of 20 g/L sodium alginate and 60 g/L polyvinyl alcohol (PVA). Through a peristaltic pump the mixture was dropped into saturated boric acid solution containing 20 g/L CaCl_2_ to form immobilized cell beads. The beads were kept at 4°C for 4 h and then washed three times with sterile deionized water. Then the bead was maintained in 5% NaCl saturated solution at 4°C for inoculation and the diameter of immobilized cell beads was 3 mm in average.

### 2.5. Fermentation Dynamics of Immobilized Whole Cell for Xylitol Production from Corncob Acid Hydrolysate Detoxified by SAA Pretreatment and Two-Step Adsorption Method

1 L of corncob acid hydrolysate detoxified by SAA and two-step adsorption was first adjusted to pH 6.0 and then added into the medium composed of yeast extract of 5 g, MgSO_4_ *·*7H_2_O of 0.4 g, KH_2_PO_4_ of 3 g, and (NH_4_)_2_SO_4_ of 5 g. After sterilization, 60 mL sterilization medium and 5.5 g immobilized whole cell were added into 150 mL Erlenmeyer flasks reactor, and batch fermentation for xylitol production was carried out under the conditions of 200 rpm and 30°C.

To evaluate the behaviors of xylitol fermentation production from corncob acid hydrolysate detoxified by SAA pretreatment and two-step adsorption, six parameters were examined such as percentage of xylose consumption (*Y*_*c*_), final xylitol concentration (*P*_*F*_), xylitol yield (*Y*), xylose uptake rate (*Q*_*s*_), and xylitol volumetric productivity (*Q*_*p*_). Xylitol yield was calculated as the function of the xylose consumption.

To investigate the operational stability of immobilized cell beads, five successive batch fermentation rounds were performed. At the end of each round, the fermented broth was unloaded, and the immobilized whole cell beads were washed with sterile deionized water and added to the fresh medium for next round. Each round lasted for 120 h with the total fermentation time of 600 h.

### 2.6. Immobilized Cell Bead Morphology Observation after Each Batch Fermentation

To evaluate the immobilized cell beads viability, scanning electron microscope (SEM, HITACHI S-3400N, Japan) was employed to observe the morphology changes of immobilized cell beads after five batch fermentation rounds. After each batch fermentation round, several immobilized cell beads were immersed in 0.5 g/L normal saline for 0.5 h, subsequently solidified for 2.5 h with the mixed solutions of 2.5% glutaraldehyde and 0.2 mol/L paraformaldehyde. The solidified beads were washed with ethanol followed by isoamyl acetate twice for 30 min each time. Then, the beads were dried by supercritical CO_2_ and cut open for morphology observation.

### 2.7. Cellulose, Xylan, and Lignin Content Determination

The cellulose and xylan contents of corncob material were determined according to the methods reported by Moore and Johnson [16]. The content of acid-insoluble lignin in corncob material was measured according to the Tappi test method T 222 om-06 (2006).

### 2.8. Sugars and Inhibitors Content Determined by HPLC and UV-Vis Spectrophotometry Analysis

The sugars contents of glucose, xylose, and arabinose in the reaction system were analyzed by HPLC (Waters 2695e, USA) equipped with refractive index detection detector and Aminex HPX-87P column (300 × 7.8 mm: Bio-Rad, USA). The temperature of column and detector was set at 85°C and 30°C, respectively. The mobile phase was ultrapure water and its flow rate was fixed at 0.6 mL/min. The injection volume of sample was 10 *μ*L.

The inhibitors content of acetic acid, 5-hydroxymethylfurfural, and furfural in hydrolysate were analyzed by HPLC (Waters 2695e, USA) and equipped with Aminex HPX-87H column (300 × 7.8 mm: Bio-Rad, USA) and refractive index detection detector. The temperature of column and detector was set at 65°C and 30°C, respectively. 5 mM sulfuric acid was used as the mobile phase and its flow rate was 0.6 mL/min. The injection volume of sample was 10 *μ*L.

The inhibitor content of total phenolic compounds in hydrolysate was determined by UV spectrophotometry according to American Public Health Association [17].

### 2.9. Statistical Analysis

All trials were studied in triplicate and the experimental data were analyzed by the software SAS 9.0 (product of SAS Institute Inc., Cary, NC, USA). Experimental data were expressed as mean value (*x*) ± standard deviation (SD).

## 3. Results and Discussion

### 3.1. Effect of SAA Pretreatment on Cellulose, Xylan, and Lignin Contents

**Figure 1:**
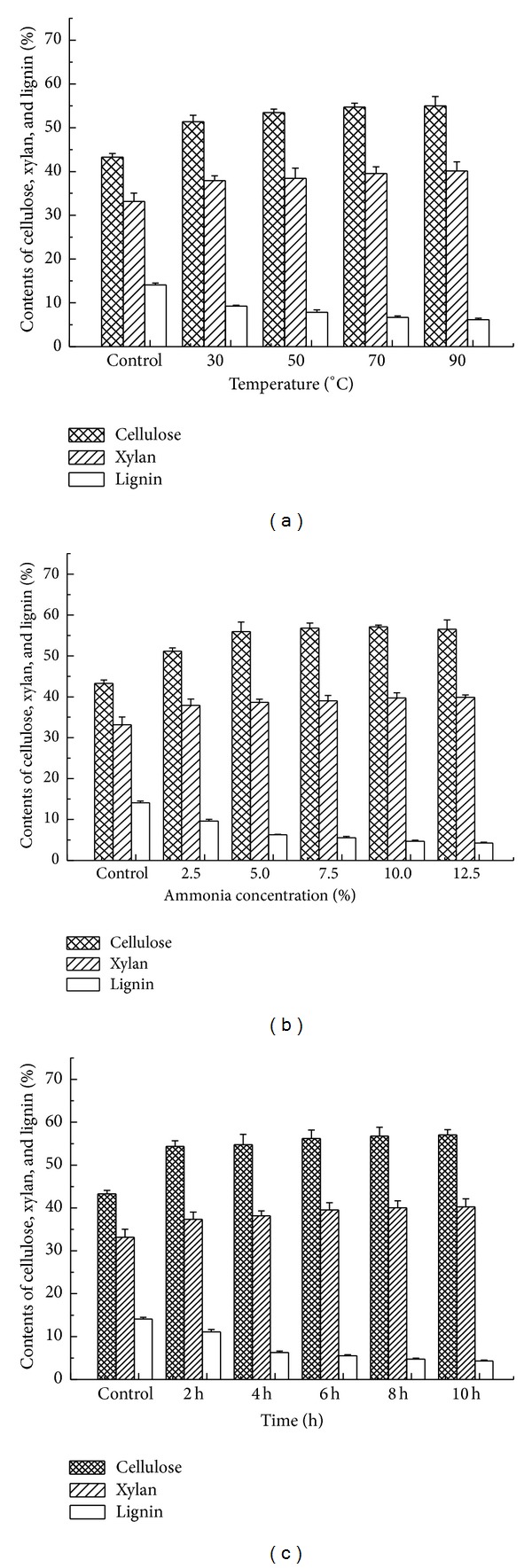
Chemical compositions of corncob with and without SAA pretreatment ((a) the ammonia concentration in aqueous solution 5%, time 6 h; (b) temperature 90°C, time 6 h; (c) temperature 70°C, the ammonia concentration in aqueous solution 10%).

The contents of cellulose, xylan, and lignin in the corncob with and without SAA pretreatment were examined and the experimental data are depicted in Figure 1.

It can be seen from Figure 1 that the corncob solid recovery was ranging from 80 to 69% with a decrease tendency mainly due to the decrease of lignin content. Compared with the control, the contents of cellulose and xylan increased for the corncob detoxified by SAA pretreatment, and 90–97% cellulose was obtained for SAA pretreatment, which is in good agreement with the results in literature [18].

Figure 1 shows that 82–93% xylan is achieved for SAA pretreatment under the variable tested conditions. Kim and Lee [19] reported that about 15% xylan of corn stover was reduced after 10 days of ammonia soaking pretreatment. It was demonstrated that more than 50% hemicellulose was reduced after ammonia soaking pretreatment [19]. Therefore, higher xylan content was observed after the corncob was pretreated by SAA in this work.

Compared with the control, Figure 1 also reveals that 39–79% lignin of corncob is removed after SAA pretreatment within 10 h. The removal of lignin is helpful to improve cellulose digestibility. This observation agreed well with the data in literatures. Kim and Lee [19] reported that 55.8% delignification after 10 d and 73.5% delignification after 60 d were obtained after 29.5 wt% ammonia soaking pretreatment at room temperature. Gao et al. [20] considered that the delignification of wheat straw was observed from 24.8% to 19.6% after 60 h pretreatment via 28–30% (w/w) ammonia soaking at 50°C. It was pointed out that 40–50% (w/w) lignin was removed off in ammonia soaking pretreatment. Ko et al. [18] presented that 37–60% (w/w) lignin was removed via SAA pretreatment. Compared with delignification via SAA pretreatment in literatures, higher delignification of corncob was achieved in this work within a bit shorter periods pretreatment.

### 3.2. Influence of SAA Pretreatment on Recovery of Sugars in the Corncob Acid Hydrolysate

The effect of SAA pretreatment was investigated on the recovery of three monomeric sugars including xylose, glucose, and arabinose in the corncob acid hydrolysate and the data are shown in Table 1.

From Table 1, it can be seen that xylose was the main monosaccharide in the hydrolysates with a small fraction of glucose and arabinose. Compared with the control, lower glucose and higher arabinose content were obtained for SAA pretreatment samples. These observations were in agreement with the results by Gao et al. [20]. Although xylose content in hydrolysate remained almost stable with SAA pretreatment, however, compared with the control, the xylan hydrolysis rate decreased by about 10% after SAA pretreatment. The reasonable explanation was probably that the structure of xylan was modified after SAA pretreatment. Liu et al. [21] suggested that xylose recovery rate could be enhanced by prolonging the reaction time and temperature of acid hydrolysis.

### 3.3. Influence of SAA Pretreatment on Inhibitors Content in the Corncob Acid Hydrolysate

**Figure 2:**
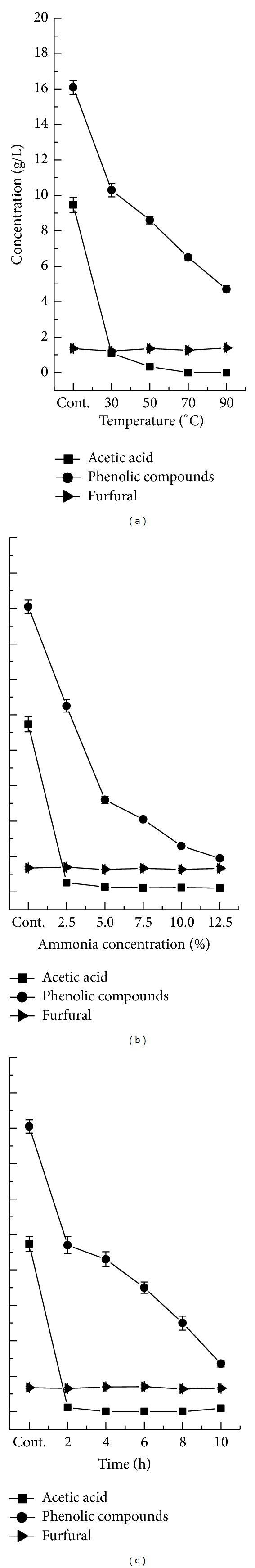
Influence of SAA conditions on the inhibitors concentration in the corncob acid hydrolysate ((a) the ammonia concentration in aqueous solution 5% and time 6 h; (b) temperature 90°C and time 6 h; (c) temperature 70°C and the ammonia concentration in aqueous solution 10%).

The effect of SAA pretreatment on the inhibitors content in the hydrolysates of corncob was evaluated and the data are shown in Figure 2.

Compared with the control, it was obviously observed that acetic acid and phenolic compounds in the hydrolysates were successfully removed after SAA pretreatment. Under the conditions of over 30°C and 2 h, inhibitor of acetic acid in corncob acid hydrolysate was fully undetected by SAA pretreatment. The inhibitors of phenolic compounds in corncob acid hydrolysate decreased more than 83% by SAA pretreatment. However, the furfural inhibitor in hydrolysate remained nearly stable with and without SAA pretreatment. Vacuum concentration processing can be usually used to remove off furfural inhibitor in the corncob acid hydrolysate. Another inhibitor of 5-hydroxymethylfurfural in the hydrolysate was not detected due to its very low concentration. In conclusion, SAA pretreatment is very helpful to lower down the inhibitors content in the corncob acid hydrolysate.

### 3.4. Comparison of the Effect of SAA Pretreated and Two-Step Adsorption Detoxification on Corncob Acid Hydrolysis

Experiments were carried out to compare the hydrolysis characteristics of corncob detoxified by SAA and two-step adsorption method, and the results are shown in Table 2.

It could be seen from Table 2 that 98.12 ± 1.94 g xylose per liter hydrolysate was obtained via SAA pretreatment, while 93.64 ± 1.55 g xylose per liter hydrolysate was achieved by two-step adsorption detoxification. The content of arabinose in hydrolysate via SAA pretreatment was higher than that via adsorption method. On the other hand, compared with two-step adsorption detoxification, the inhibitors formation in the hydrolysate was lower using SAA pretreatment detoxification. In view of SAA pretreatment, acetic acid and furfural compound were not detected in acid hydrolysate, and phenolic compounds content was about 6.75 g/L in the acid hydrolysate. For adsorption detoxification, the content of acetic acid and phenolic compounds was 5.72 g/L and 10.85 g/L, respectively. It indicated that higher xylose and arabinose could be obtained in the hydrolysate via SAA pretreatment than via adsorption detoxification.

### 3.5. Behavior of Xylitol Production by Repeated-Batch Fermentation from Corncob Hydrolysate by Immobilized Whole Cell of* C. tropicalis*

**Figure 3:**
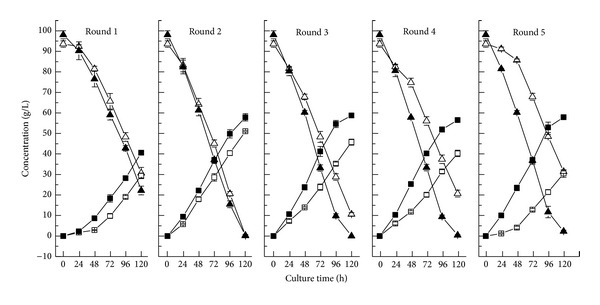
Xylose consumption and xylitol yield in repeated batch fermentation for xylitol production by immobilized whole cell from corncob acid hydrolysate via SAA pretreatment and two-step adsorption detoxification (▲△ residual xylose, ■□ xylitol yield; solid symbols refer to SAA pretreatment method, and hollow symbols refer to adsorption detoxification).

To demonstrate the operational stability of immobilized whole cell beads, five successive batch fermentation rounds were performed for xylitol production from corncob acid hydrolysate detoxified by SAA and adsorption treatment. Each round lasted for 120 h with the total fermentation time of 600 h. At the end of each round, the immobilized whole cell was introduced to the fresh medium for next round. The results are shown in Figure 3.

From Table 3, it could be obviously observed that the xylose consumed (*Y*_*c*_) was 77.4 ± 0.11% in the first round batch fermentation for xylitol production from corncob acid hydrolysate via SAA pretreatment by immobilized* C. tropicalis* cell, and almost all xylose (more than 99%) was consumed in the continuing next 4-round batch fermentation. In view of adsorption detoxification processing, the *Y*_*c*_ was 67.2 ± 0.77% in the first round batch fermentation, and it was almost consumed completely in the second round batch fermentation; after that, *Y*_*c*_ decreases by 10% and 66.5 ± 1.85% xylose was consumed in the fifth round batch fermentation. The reasonable explanation was that immobilized* C. tropicalis* cell did not adapt to the conditions with high xylose content in the first round batch fermentation; after preculture, the activity of immobilized* C. tropicalis* cell was enhanced in the 2nd, 3rd, 4th, and 5th batches. However, owing to the inhibitors existence in adsorption detoxification processing, the activity of immobilized* C. tropicalis* cell was inhibited. So the  *Y*_*c*_ decreases in 3rd, 4th, and 5th batches fermentation for adsorption detoxification processing. This phenomenon agreed well with that reported by Cunha et al. [22], who demonstrated that the lowest xylitol yield was obtained in the first round for repeated batch fermentation using immobilized* Candida guilliermondii* to produce xylitol from sugarcane bagasse acid hydrolysate.

The other fermentation parameters, such as final xylitol concentration each round (*P*_*F*_), yield of xylitol based on consumed xylose (*Y*), xylose uptake rate (*Q*_*s*_), and xylitol volumetric productivity (*Q*_*p*_), are more satisfactory in batch fermentation for xylitol production from corncob hydrolysate via SAA pretreatment than that by adsorption detoxification. All these data are depicted in Table 3.

It could be revealed from Figure 3 and Table 3 that higher performance properties of the immobilized* C. tropicalis* whole cell were observed for xylitol fermentation production from corncob acid hydrolysate detoxified by SAA than by adsorption treatment in the five successive batch fermentation rounds. After the fifth round, about 60 g xylitol/L was achieved for SAA pretreatment and only about 30 g xylitol/L for adsorption detoxification. It was noticed that the xylitol yield was lower in the first batch fermentation.

### 3.6. Morphology of Immobilized Cell Beads after Five Batch Fermentation Rounds for Xylitol Production

**Figure 4:**
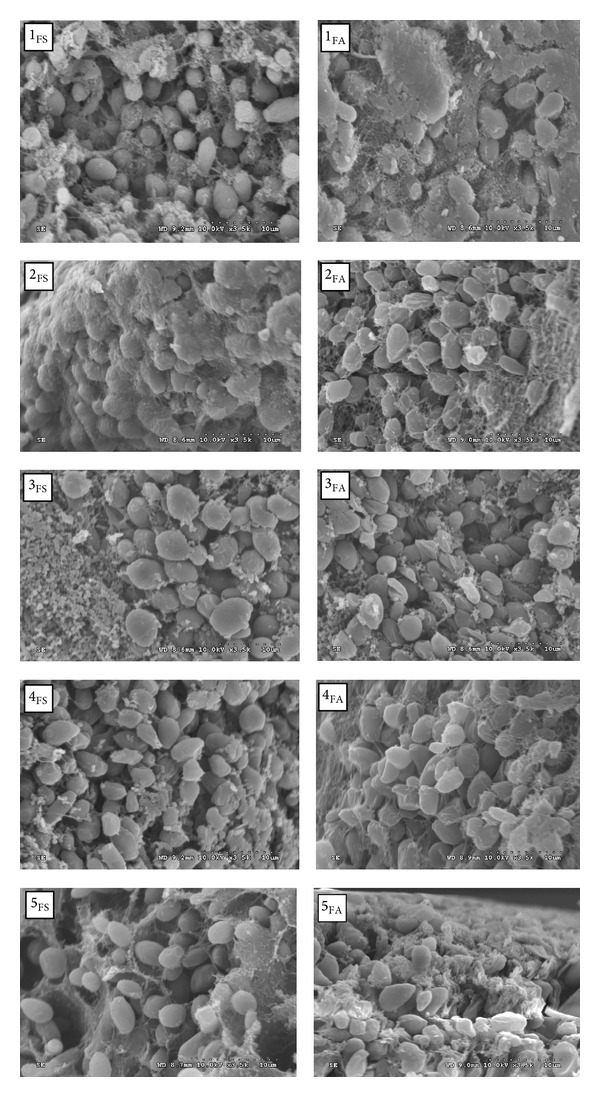
The morphology of immobilized cell bead after five batch fermentation rounds (FS: fermentation of hydrolysate detoxified by SAA pretreatment; FA: fermentation of hydrolysate detoxified by two-step adsorption method. The digit number of 1, 2, 3, 4, and 5 means batch fermentation round).

In order to further understand the viability of the immobilized cell beads during the five-round fermentation, the morphology of the immobilized cells was observed by SEM and the results are shown in Figure 4.

From Figure 4, it could be seen that the cell beads kept a complete round shape after five-round repeated fermentation. It was estimated that* ca.* 90% of cells kept activity for SAA pretreatment processing, while 84% of cells kept activity for two-step absorption processing in the two-round repeated usage. It suggested that the cell biomass concentration could have changed between one and another from 10% to 16%. If the beads were saturated in biomass previously to their use in the batch fermentation, the initial xylose conversion was increased but the final cell concentration was little affected after five-round repeated usages. The deformation percentage of immobilized whole cell was less in the hydrolysate via SAA pretreatment than via two-step adsorption. The reasonable explanation was the fact that lower inhibitors formation was found in the hydrolysate via SAA pretreatment. It was demonstrated that toxification effect of inhibitors in the reaction system increased with the concentration of inhibitors, and there was a synergistic interaction effect between inhibitors [7, 23]. In addition, Cheng et al. [9] revealed that final xylitol yield in the broth was less than 72% when acetic acid reached 4 g/L in the reaction system at pH 4.5.

## 4. Conclusions

SAA pretreatment of corncob is a novel strategy to reduce the inhibitors formation with merits of high delignification and efficient removal of acetyl group bounded to oligomer with high recoveries of cellulose and xylan. The immobilized cell beads of* C. tropicalis* retained higher viability in the hydrolysate obtained via SAA than that detoxified by the two-step adsorption. Fermentation dynamics of immobilized whole cell for xylitol production from corncob acid hydrolysate after detoxified by SAA pretreatment and two-step adsorption method was compared, and it was shown that SAA would be a promising pretreatment method to delignify and increase the accessibility of hydrolytic enzymes to cellulose. Repeated-batch fermentation showed that the immobilized cell beads presented high operational stability for xylitol production.

## Figures and Tables

**Table 1:** Influence of SAA conditions on recovery of sugars and xylan hydrolysis.

Conditions	Glucose (g/L)	Xylose (g/L)	Arabinose (g/L)	Xylan hydrolysis rate (%)
Control	1.47 ± 0.07	33.42 ± 1.33	2.06 ± 0.11	77.04 ± 0.13
Temperature^a^ (°C)				
30	0.43 ± 0.05	32.96 ± 1.07	2.47 ± 0.14	67.31 ± 0.12
50	0.32 ± 0.02	32.41 ± 1.04	3.26 ± 0.18	66.82 ± 0.11
70	0.32 ± 0.03	32.89 ± 1.93	3.74 ± 0.12	66.71 ± 0.14
90	0.45 ± 0.04	33.06 ± 0.97	4.11 ± 0.16	67.03 ± 0.11
Ammonia content^b^ (%)				
2.5	0.27 ± 0.03	31.90 ± 1.46	3.16 ± 0.16	66.61 ± 0.15
5.0	0.34 ± 0.03	32.47 ± 0.88	3.63 ± 0.10	66.70 ± 0.14
7.5	0.44 ± 0.02	32.30 ± 1.53	3.89 ± 0.08	66.80 ± 0.11
10	0.45 ± 0.03	32.46 ± 0.68	3.98 ± 0.13	66.09 ± 0.16
12.5	0.35 ± 0.02	32.88 ± 0.92	3.88 ± 0.21	66.32 ± 0.31
Time^c^ (h)				
2	0.35 ± 0.05	32.42 ± 1.48	3.41 ± 0.19	69.04 ± 0.22
4	0.46 ± 0.02	32.58 ± 1.71	3.84 ± 0.22	68.71 ± 0.33
6	0.43 ± 0.04	32.76 ± 0.75	3.91 ± 0.15	66.73 ± 0.12
8	0.45 ± 0.02	32.73 ± 1.69	4.01 ± 0.24	66.1 ± 0.24
10	0.50 ± 0.03	33.06 ± 1.87	4.12 ± 0.28	66.5 ± 0.31

^a^Ammonia concentration in aqueous solution 5% and time 6 h.

^b^Temperature 90°C and time 6 h.

^c^Temperature 70°C and ammonia concentration in aqueous solution 10%.

**Table 2:** Characteristics of sugars and inhibitors in corncob acid hydrolysate via SAA pretreatment and two-step adsorption detoxification.

Hydrolysate	Compositions					
	Glucose /(g/L)	Xylose /(g/L)	Arabinose /(g/L)	Acetic acid /(g/L)	Furfural /(g/L)	Phenolic compounds /(g/L)
Original concentration of hydrolysate	1.47 ± 0.07	33.42 ± 1.33	2.06 ± 0.11	9.47 ± 0.43	1.362 ± 0.06	16.1 ± 0.38
Detoxified by SAA^a^ and concentrated^b^	1.61 ± 0.10	98.12 ± 1.94	12.46 ± 1.65	ND^c^	ND	6.75 ± 0.38
Concentrated^b^ and detoxified by adsorption	4.63 ± 0.27	93.64 ± 1.55	5.55 ± 0.41	5.72 ± 0.34	ND	10.85 ± 0.42

^a^SAA pretreatment performed 10 h at 70°C using 10.0% aqueous ammonia.

^b^The hydrolysates were concentrated 3-fold.

^c^ND: not detected.

**Table 3:** Behaviour of repeated-batch fermentation for xylitol production by immobilized whole cell from corncob acid hydrolysate via SAA pretreatment.

Parameters	Repeated-batch fermentation									
	Round 1		Round 2		Round 3		Round 4		Round 5	
	DS	DA	DS	DA	DS	DA	DS	DA	DS	DA
*Y*_*c*_/%	77.4 ± 0.11	67.2 ± 0.77	99.8 ± 0.72	99.7 ± 0.72	100.0 ± 1.00	88.7 ± 0.45	99.6 ± 1.65	77.9 ± 0.14	99.7 ± 1.37	66.5 ± 1.85
*P*_*F*_/g*·*L^−1^	40.58 ± 0.56	29.42 ± 1.11	57.84 ± 1.78	51.07 ± 0.36	58.71 ± 1.03	45.77 ± 1.46	56.50 ± 0.94	40.27 ± 1.58	57.90 ± 0.37	30.45 ± 1.85
*Y*/g*·*g^−1^	0.53 ± 0.02	0.47 ± 0.01	0.59 ± 0.01	0.55 ± 0.00	0.60 ± 0.02	0.55 ± 0.01	0.59 ± 0.01	0.55 ± 0.02	0.60 ± 0.00	0.50 ± 0.00
*Q*_*s*_/g*·*L^−1^ *·*h^−1^	0.63 ± 0.00	0.52 ± 0.01	0.82 ± 0.01	0.78 ± 0.01	0.82 ± 0.01	0.69 ± 0.00	0.81 ± 0.01	0.61 ± 0.01	0.80 ± 0.00	0.52 ± 0.02
*Q*_*p*_/g*·*L^−1^ *·*h^−1^	0.34 ± 0.00	0.25 ± 0.01	0.48 ± 0.01	0.43 ± 0.00	0.49 ± 0.01	0.38 ± 0.01	0.47 ± 0.01	0.34 ± 0.01	0.48 ± 0.00	0.25 ± 0.02

DS: detoxification via SAA pretreatment; DA: detoxification via adsorption.

*Y*_*c*_ *:* percentage of xylose consumption each round; *P*_*F*_: final xylitol concentration each round; *Y*: yield of xylitol based on consumed xylose; *Q*_*s*_: xylose uptake rate; *Q*_*p*_: xylitol volumetric productivity.
